# Supplementary material for: NMDAR-mediated modulation of gap junction circuit regulates olfactory learning in C. elegans
Source: Nat Commun. 2020 Jul 10;11:3467. doi: 10.1038/s41467-020-17218-0 (PMC7351742; doi:10.1038/s41467-020-17218-0)
Supplement: Supplementary file 1 — Supplementary Information [file 41467_2020_17218_MOESM1_ESM.pdf]

Supplementary Materials for Choi et al.

NMDAR-mediated modulation of gap junction circuit regulates olfactory learning in *C. elegans*

*Correspondence to: yzhang@oeb.harvard.edu

Supplementary Figures 1 through 14 and Supplementary Methods.

Supplementary Fig. 1

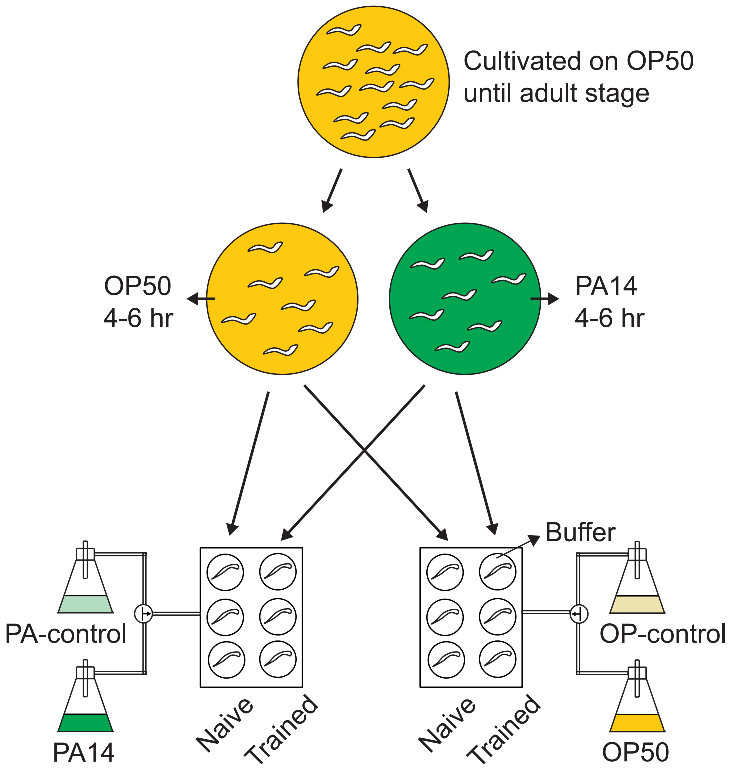

Supplementary Fig. 1. The schematic diagram showing the procedure for aversive training with PA14 and droplet assay for olfactory learning. *C. elegans* hermaphrodites are cultivated under the standard condition on *E. coli* OP50 until the adult stage and divided into two groups. The trained animals are fed on *P. aeruginosa* PA14 for 4 – 6 hours and the naive control animals are fed on OP50 in parallel. Both the naive animals and the trained animals are tested for their preference between the undiluted PA14 bacteria culture (PA14) and the 4-fold diluted PA14 bacteria culture (PA-control), as well as their preference between the undiluted OP50 bacteria culture (OP50) and the 4-fold diluted OP50 bacteria culture (OP-control), using a droplet assay where the smell of a bacteria culture is delivered using an air stream to the individual animals swimming in the droplets of buffer in an enclosed chamber. Two air streams delivering different odorant stimuli alternate every 30 seconds and the behavior of the worms is recorded and scored using an automated device (Methods).

Supplementary Fig. 2

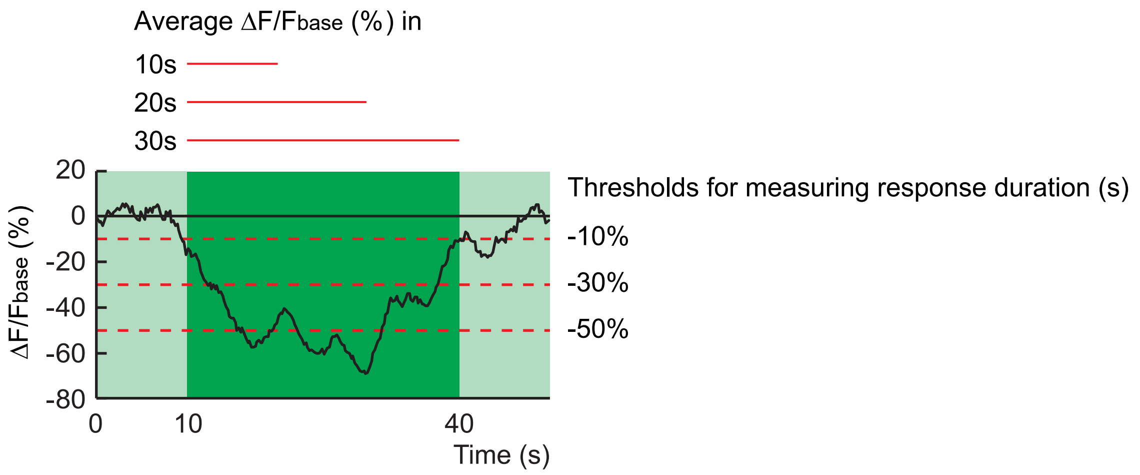

Supplementary Fig. 2. Analyses of response amplitude and response duration on calcium imaging traces. Average response amplitude of GCaMP3 signal, average $\Delta F/F_{base}$ (%), upon stimulation is measured for three different durations (10s, 20s or 30s) for AIB, AVA and RIM. Response duration is measured using three different thresholds ($\Delta F/F_{base}$ % < -10%, -30% or -50%).

Supplementary Fig. 3

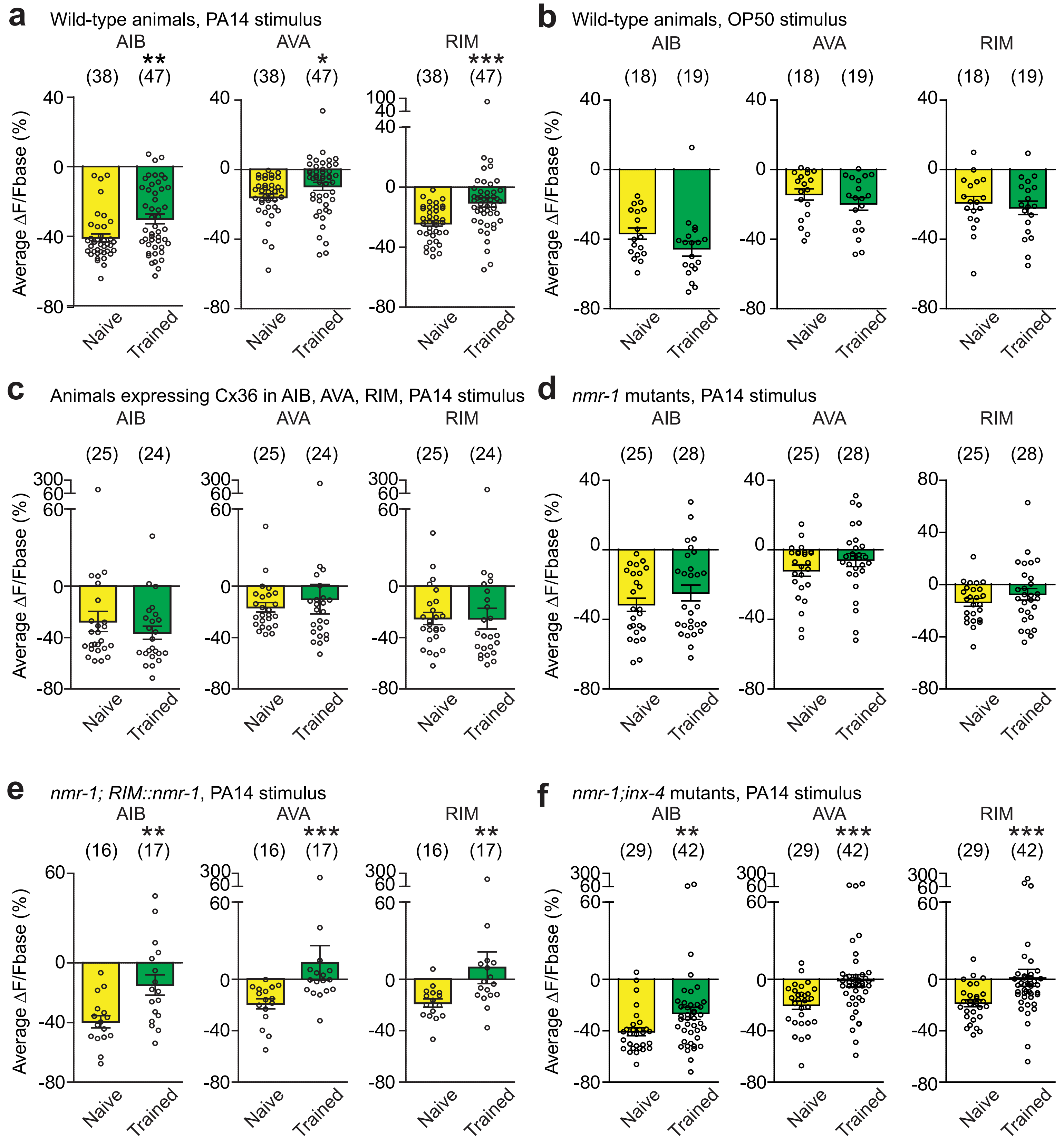

Supplementary Fig. 3. Average response amplitude of PA14 or OP50-evoked GCaMP3 signals of AIB, AVA and RIM in the first 10s of stimulation. (**a - f**) In the first 10s of stimulation, average response amplitude of PA14-evoked (**a, c – f**) or OP50-evoked (**b**) GCaMP3 signals in wild-type, transgenic and mutant animals. Parentheses contain the numbers of animals examined over 9 (**a**) or 3 (**b**) or 7 (**c**) or 6 (**d**) or 3 (**e**) or 5 (**f**) independent experiments, naive and trained animals are compared using two-sided Mann-Whitney U test or two-sided Welch's t-test, asterisks indicate significant difference, *** $p < 0.001$, ** $p < 0.01$, * $p < 0.05$, mean ± s.e.m., circles indicate individual data points. The p values in each panel, from left to right, are: **a**, p=0.0069, 0.012, <0.0001; **b**, p=0.11, 0.27, 0.60; **c**, p=0.40, 0.60, 0.38; **d,** p=0.33, 0.22, 0.25; **e,** p=0.0044, 0.0007, 0.0025; **f,** p=0.0078, 0.0006, 0.0007. Source data are provided as a Source Data file.

Supplementary Fig. 4

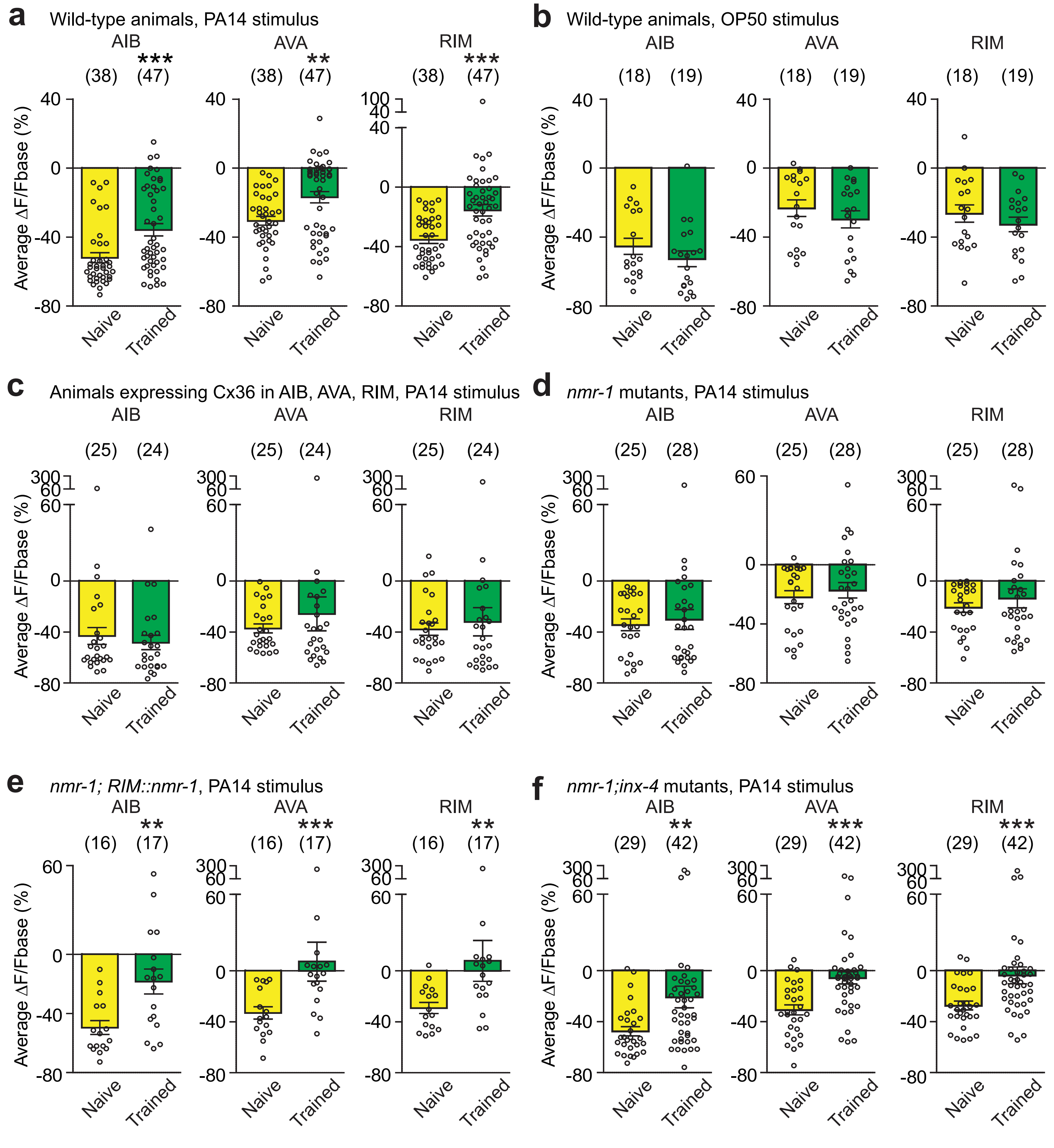

Supplementary Fig. 4. Average response amplitude of PA14 or OP50-evoked GCaMP3 signals of AIB, AVA and RIM in the first 20s of stimulation. (**a - f**) In the first 20s of stimulation, average response amplitude of PA14-evoked (**a, c – f**) or OP50-evoked (**b**) GCaMP3 signals in wild-type, transgenic and mutant animals. Parentheses contain the numbers of animals examined over 9 (**a**) or 3 (**b**) or 7 (**c**) or 6 (**d**) or 3 (**e**) or 5 (**f**) independent experiments, naive and trained animals are compared using two-sided Mann-Whitney U test or two-sided Welch's t-test, asterisks indicate significant difference, *** $p < 0.001$, ** $p < 0.01$, * $p < 0.05$, mean ± s.e.m., circles indicate individual data points. The p values in each panel, from left to right, are: **a**, p=0.0005, 0.0018, <0.0001; **b**, p=0.25, 0.36, 0.34; **c,** p=0.48, 0.84, 0.64; **d**, p=0.65, 0.85, 0.98; **e**, p=0.0034, 0.0008, 0.0019; **f**, p=0.0023, <0.0001, 0.0004. Source data are provided as a Source Data file.

Supplementary Fig. 5

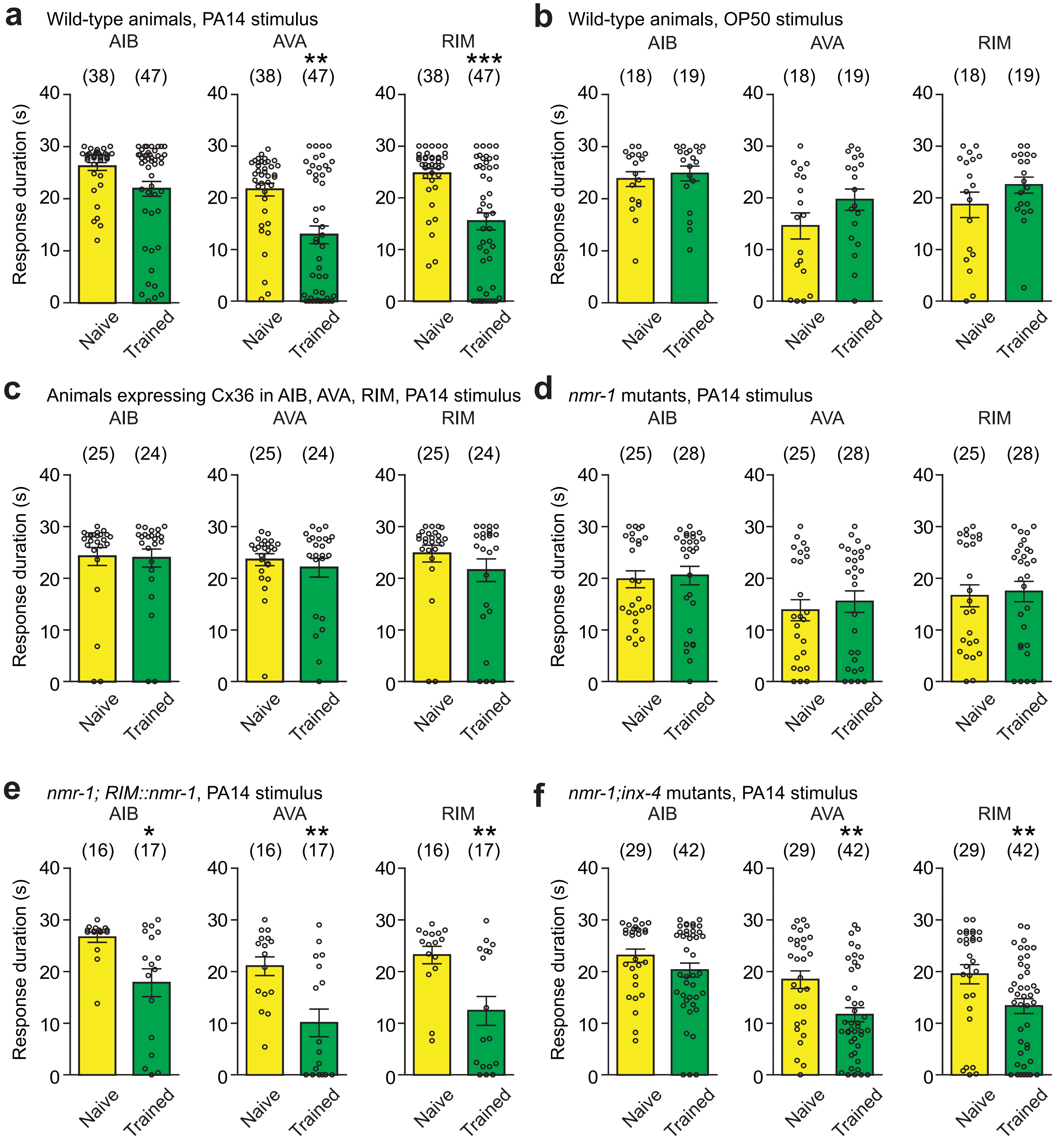

Supplementary Fig. 5. Response duration of PA14 or OP50-evoked GCaMP3 signals with $\Delta F/F_{base}$ < - 10% in AIB, AVA and RIM. (**a - f**) Response duration of PA14-evoked (**a**, **c – f**) or OP50-evoked (**b**) GCaMP3 signals with $\Delta F/F_{base}$ < - 10% in wild-type, transgenic and mutant animals. Parentheses contain the numbers of animals examined over 9 (**a**) or 3 (**b**) or 7 (**c**) or 6 (**d**) or 3 (**e**) or 5 (**f**) independent experiments, naive and trained animals are compared using two-sided Mann-Whitney U test or two-sided Welch's t-test, asterisks indicate significant difference, *** $p < 0.001$, ** $p < 0.01$, * $p < 0.05$, mean ± s.e.m., circles indicate individual data points. The p values in each panel, from left to right, are: **a**, p=0.077, 0.0018, 0.0001; **b**, p=0.46, 0.13, 0.20; **c**, p=0.96, 0.62, 0.77; **d**, p=0.92, 0.73, 0.97; **e**, p=0.015, 0.0048, 0.0038; **f**, p=0.14, 0.0027, 0.0034. Source data are provided as a Source Data file.

Supplementary Fig. 6

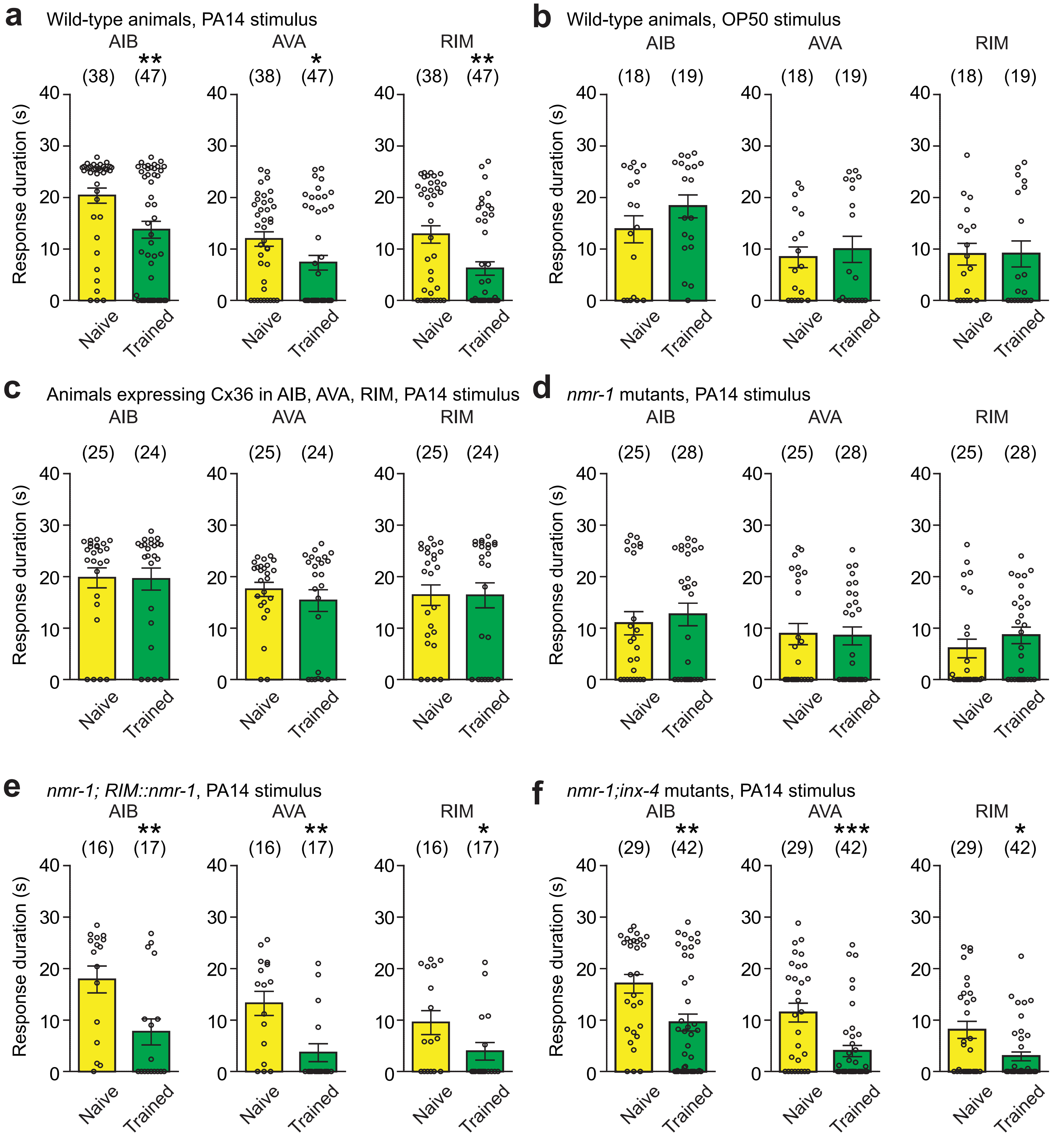

Supplementary Fig. 6. Response duration of PA14 or OP50-evoked GCaMP3 signals with ΔF/F$_{base}$ < - 50% in AIB, AVA and RIM. (**a - f**) Response duration of PA14-evoked (**a**, **c – f**) or OP50-evoked (**b**) GCaMP3 signals with ΔF/F$_{base}$ < - 50% in wild-type, transgenic and mutant animals. Parentheses contain the numbers of animals examined over 9 (**a**) or 3 (**b**) or 7 (**c**) or 6 (**d**) or 3 (**e**) or 5 (**f**) independent experiments, naive and trained animals are compared using two sided Mann-Whitney U test, asterisks indicate significant difference, *** $p < 0.001$, ** $p < 0.01$, * $p < 0.05$, mean ± s.e.m., circles indicate individual data points. The p values in each panel, from left to right, are: **a**, p=0.0073, 0.023, 0.001; **b**, p=0.13, 0.77, 0.90; **c**, p=0.60, 0.79, 0.63; **d**, p=0.97, 0.80, 0.32; **e**, p=0.006, 0.0033, 0.045; **f**, p=0.0037, 0.0008, 0.012. Source data are provided as a Source Data file.

Supplementary Fig. 7

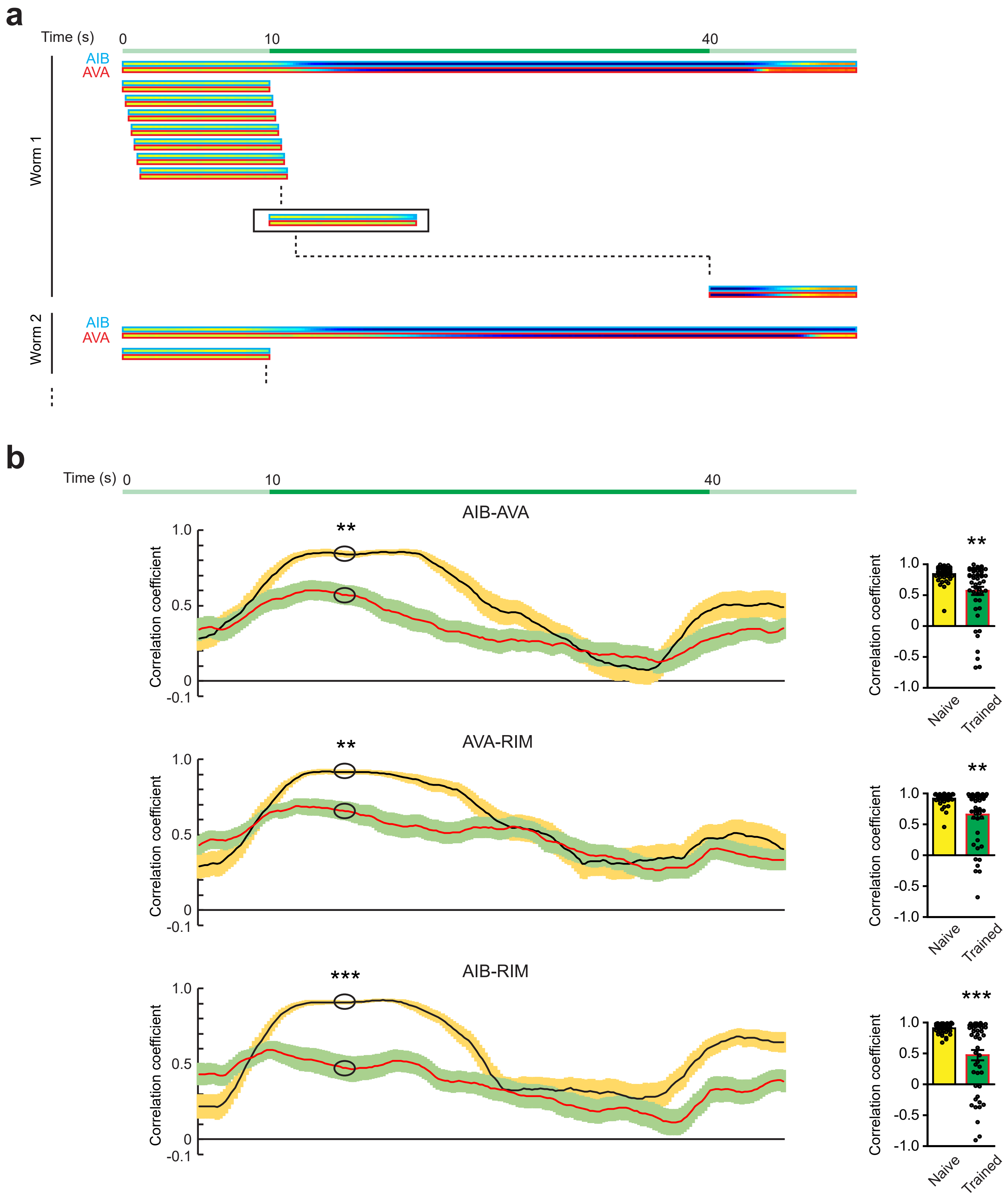

Supplementary Fig. 7. Cross-correlation analysis using a 10-second sliding window. (**a**) Schematic diagram showing that for each pair of neurons, correlation coefficients of their GCaMP3 signals are measured using a 10-second time window that slides every 0.2 second starting at 0 second and ending at 50 second. (**b**) Correlation coefficients measured using a 10-second sliding window for GCaMP3 signals of AIB, AVA and RIM in naive (n = 38) and trained (n = 47) wild-type animals shown in Fig. 1e and 1f. Lines and shades on the left are mean and s.e.m., respectively (yellow shades are for naive animals and green shades are for trained animals). Bar graphs on the right show mean and s.e.m. for the time points denoted on the left, naive and trained animals are compared using two-sided Mann Whitney U test, asterisks indicate significant difference, *** $p < 0.001$, ** $p < 0.01$, circles in the bar graphs indicate individual data points. Naive (n = 38) and trained (n = 47) wild-type animals are examined over 9 independent experiments, p values from top to bottom in bar graphs (b) are 0.0017, 0.0023, 0.0002. Source data are provided as a Source Data file.

Supplementary Fig. 8

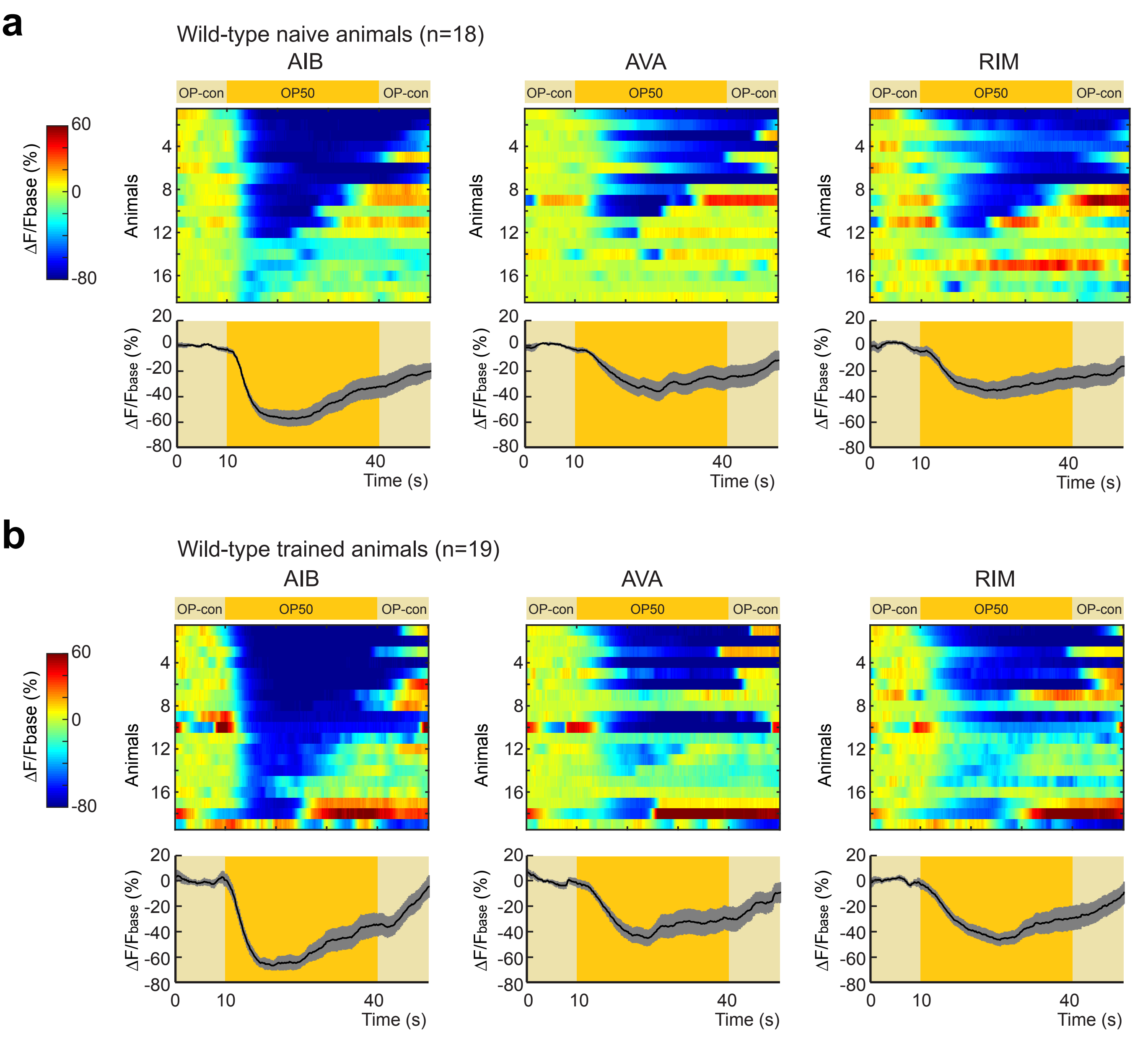

Supplementary Fig. 8. Training with PA14 does not modulate OP50-evoked responses in AIB, AVA and RIM. (**a, b**) GCaMP3 signals of AIB, AVA and RIM evoked by the switches between OP50 and OP-control (OP-con) in individual animals (top panels) and the mean values (bottom panels). GCaMP3 signals of AIB, AVA and RIM are simultaneously recorded in every animal and vertically arranged based on the response of AIB. $\Delta F = F - F_{base}$ and F_{base} is average GCaMP3 signal in the 10-second window before OP50 stimulation (Methods). Spectrums indicate the ranges of $\Delta F/F_{base}$ (%) and $\Delta F/F_{base}$ (%) are outside of indicated ranges for some frames. The solid traces and the shades in the bottom panels respectively denote mean values and s.e.m.. Parentheses contain the number of animals examined over 3 independent experiments. Source data are provided as a Source Data file.

Supplementary Fig. 9

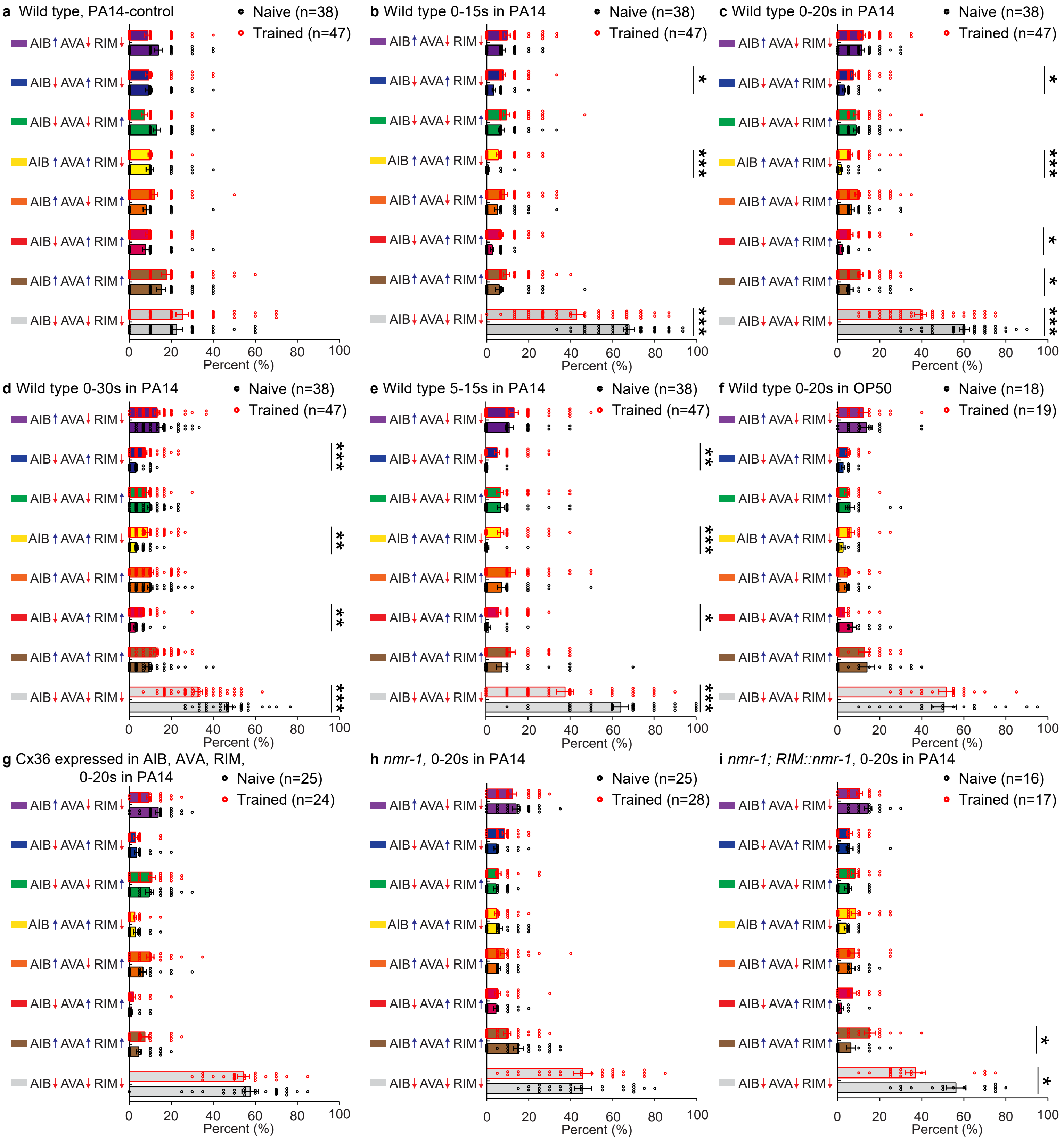

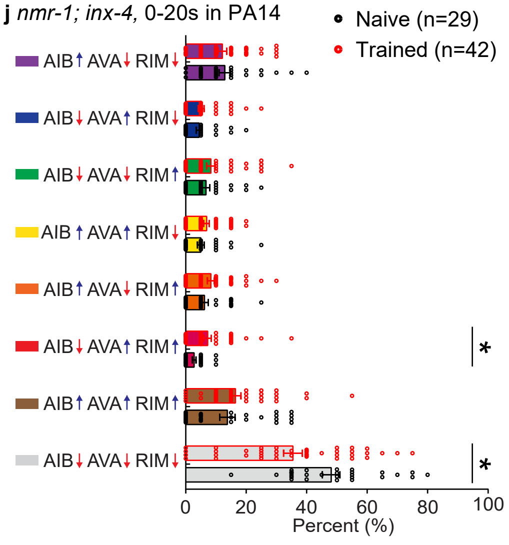

Supplementary Fig. 9. Activity patterns for RIM-circuit in naive and trained animals. (**a - j**) An upward pointing arrow or a downward pointing arrow following each neuron respectively denotes a positive or a negative value for time derivative of GCaMP3 signal of the neuron (Methods). The percentages of each activity pattern during indicated time window in naive and trained animals are compared using two-sided Welch's t-test or two-sided Mann-Whitney U test with Bonferroni correction, asterisks indicate significant difference, *** $p < 0.001$, ** $p < 0.01$, * $p < 0.05$, mean ± s.e.m..
(continued on the next page)

Parentheses contain the numbers of animals examined over 9 (**a - e**) or 3 (**f**) or 7 (**g**) or 6 (**h**) or 3 (**i**) or 5 (**j**) independent experiments, circles indicate individual data points. The Bonferroni corrected *p* values in each panel, from top to bottom, are: **a**, *p*=0.25, 1, 0.08, 1, 0.54, 1, 1, 1; **b**, *p*=1, 0.014, 1, <0.0008, 0.43, 0.092, 0.2, <0.0008; **c**, *p*=1, 0.013, 1, <0.0008, 1, 0.0496, 0.022, <0.0008; **d**, *p* =1, <0.0008, 1, 0.0064, 1, 0.0056, 0.33, <0.0008; **e**, *p*=1, 0.0064, 1, <0.0008, 0.61, 0.015, 0.13, <0.0008; **f**, *p*=1, 1, 1, 0.44, 1, 1, 1, 1; **g**, *p*=0.25, 1, 1, 1, 0.81, 1, 1, 1; **h**, *p*=1, 0.056, 1, 1, 1, 1, 1, 1; **i**, *p*=1, 1, 1, 1, 1, 0.06, 0.049, 0.047; **j**, *p*=1, 1, 1, 0.99, 1, 0.042, 1, 0.033. Source data are provided as a Source Data file.

Supplementary Fig. 10

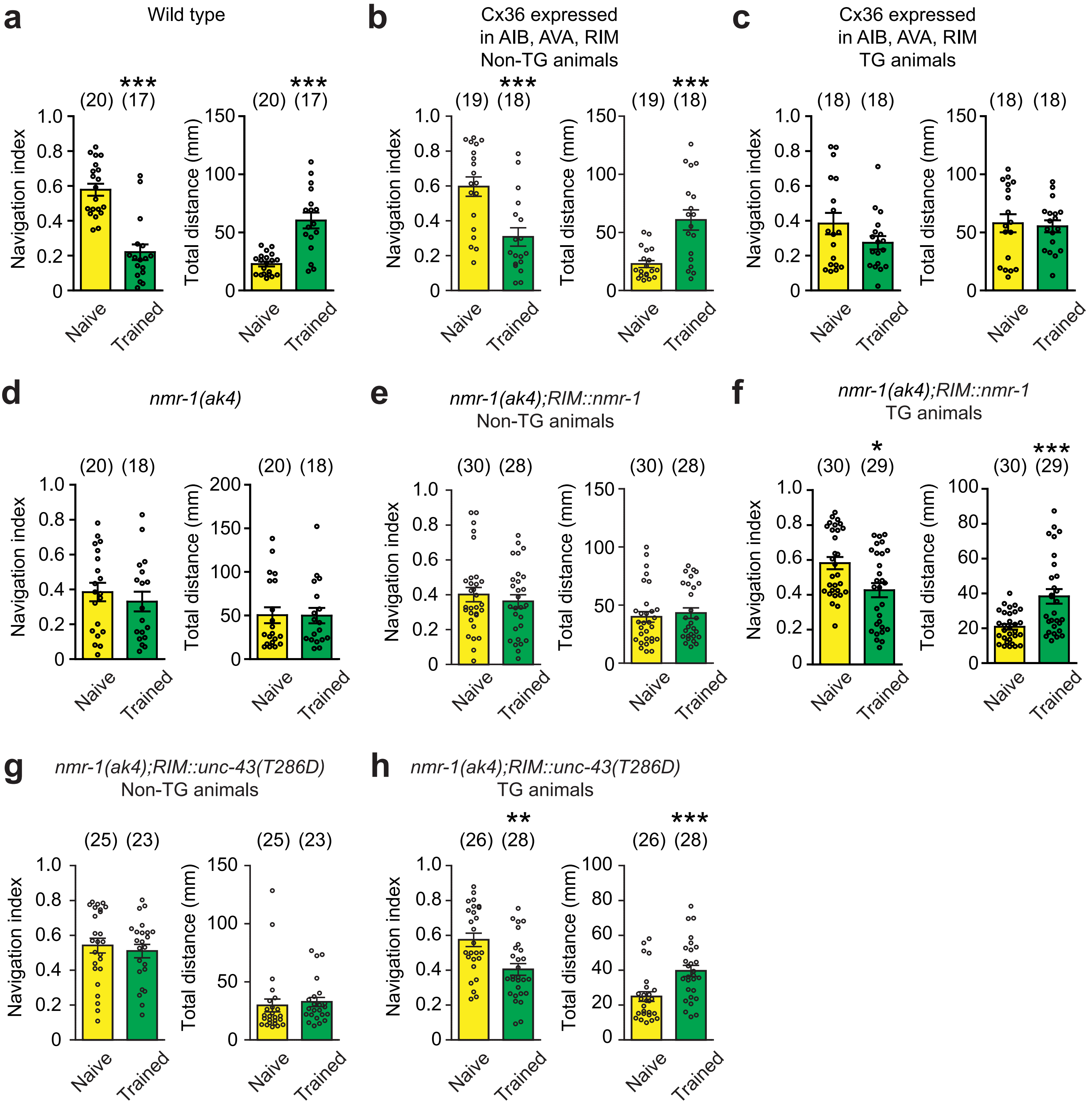

Supplementary Fig. 10. Learning of PA14 tested using a plate assay. (**a - h**) Navigation index (left panels) and travelling distance (right panels) in a plate assay where a worm navigates towards a drop of supernatant of PA14 culture in wild-type, mutants, transgenic animals (TG animals) and non-transgenic controls (non-TG animals). Training with PA14 decreases navigation index and increases traveling distance in wild type. Parentheses contain the numbers of animals examined over 3 (**a - d, g**) or 4 (**e, f, h**) independent experiments, circles indicate individual data points, naive and trained animals are compared using two-sided Welch's t-test or two-sided Mann-Whitney U test, asterisks indicate significant difference, *** $p < 0.001$, ** $p < 0.01$, * $p < 0.05$, mean ± s.e.m.. The p values in each panel, from left to right, are: **a**, p<0.0001, <0.0001; **b**, p=0.0005, 0.0006; **c**, p=0.14, 0.78; **d**, p=0.49, 0.90; **e**, p=0.49, 0.66; **f**, p=0.011, 0.0008; **g**, p=0.59, 0.079; **h**, p=0.0017, 0.0006. Source data are provided as a Source Data file.

Supplementary Fig. 11

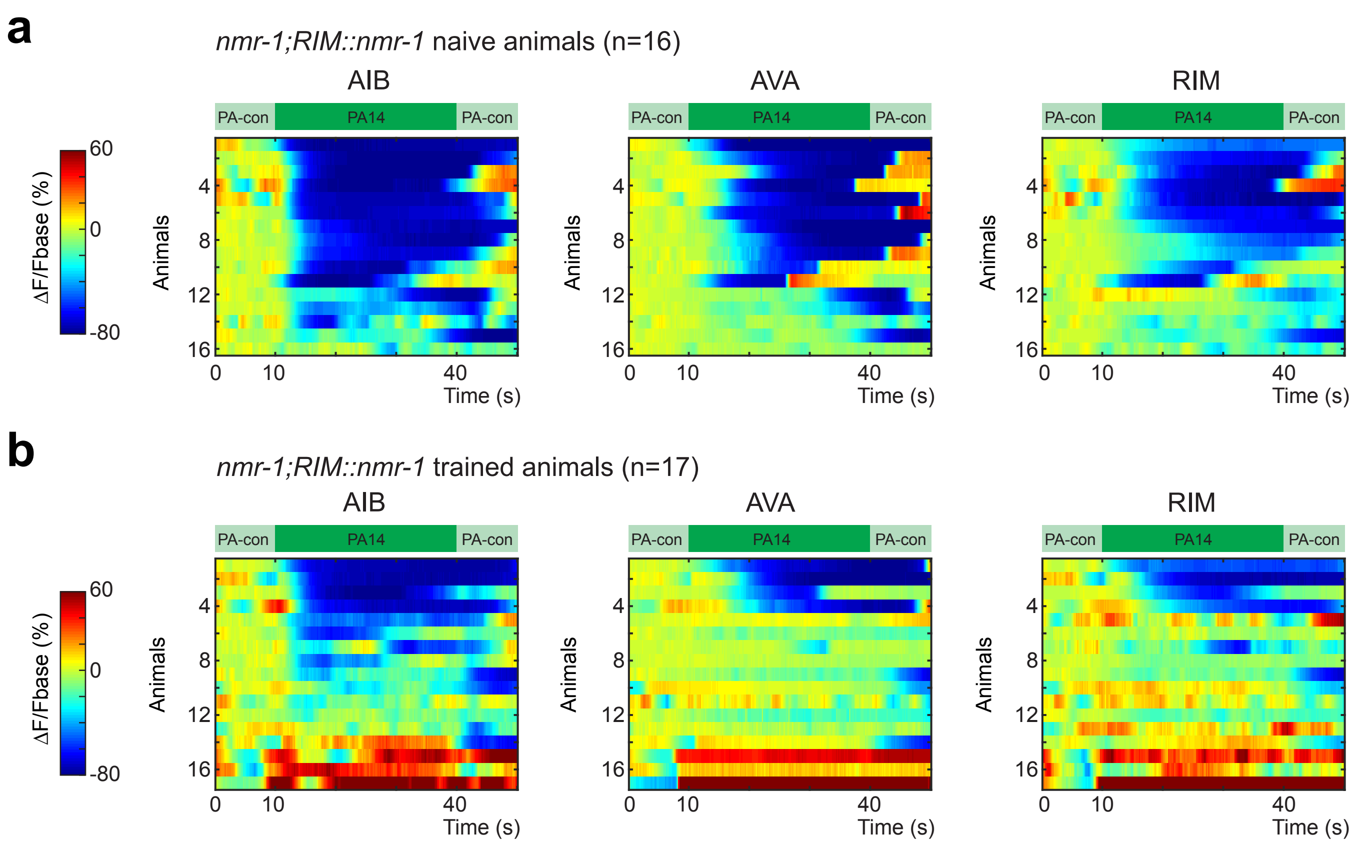

Supplementary Fig. 11. *nmr-1* acts in RIM to regulate training-dependent modulation of RIM-circuit. (**a, b**) GCaMP3 signals of AIB, AVA and RIM evoked by switches between PA14 and control (PA-con) in individual naive (**a**) and trained (**b**) transgenic animals expressing wild-type *nmr-1* cDNA in RIM in *nmr-1(ak4)* mutants. GCaMP3 signals of RIM, AIB and AVA are simultaneously recorded in each animal and vertically arranged based on GCaMP3 signals of AIB. $\Delta F = F - F_{base}$ and F_{base} is average GCaMP3 signal in the 10-second window before PA14 stimulation (Methods). Spectrums indicate ranges of $\Delta F/F_{base}$ (%) and $\Delta F/F_{base}$ (%) is outside of indicated ranges for some frames. Parentheses contain the numbers of animals examined over 3 independent experiments. Source data are provided as a Source Data file.

Supplementary Fig. 12

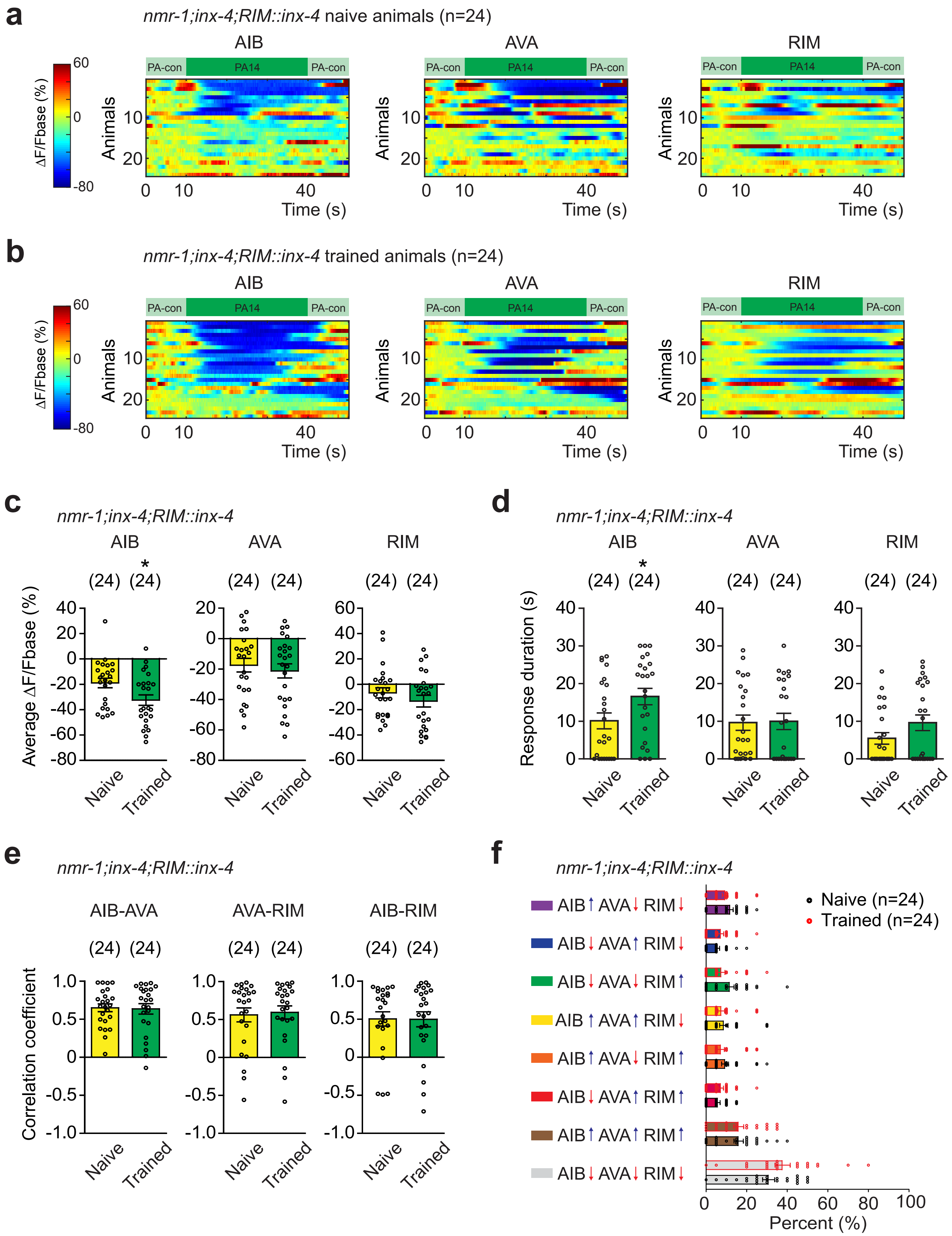

Supplementary Fig. 12. Expressing *inx-4* in RIM reverses the suppressing effect of mutating *inx-4* on *nmr-1* mutant animals. (**a, b**) GCaMP3 signals of AIB, AVA and RIM evoked by PA14 and control in individual naive (**a**) and trained (**b**) transgenic animals expressing wild-type *inx-4* cDNA in RIM in *nmr-1;inx-4* double mutants. GCaMP3 signals of RIM, AIB and AVA are simultaneously recorded in each animal and vertically arranged based on GCaMP3 of AIB. (continued on next page).

$\Delta F = F - F_{base}$ and F_{base} is average GCaMP3 signal in the 10-second window before PA14 stimulation (Methods). Spectrums indicate the ranges of $\Delta F/F_{base}$ (%) and $\Delta F/F_{base}$ (%) is outside of indicated ranges for some frames. **(c - f)** Average response amplitude during 30s exposure to PA14 (**c**), response duration for $\Delta F/F_{base}$ (%) < -30% (**d**), correlation coefficients during 30s PA14 stimulation (**e**), and percentage of each activity pattern for the first 20s of PA14 exposure (**f**) for PA14-evoked GCaMP3 signals in AIB, AVA and RIM in *nmr-1;inx-4* double mutants expressing wild-type *inx-4* cDNA in RIM. In **f**, an upward pointing or downward pointing arrow following each neuron respectively denotes a positive value or a negative value for time derivative of GCaMP3 signal of the neuron (Methods). Naive and trained animals are compared using two-sided Welch's *t*-test or two-sided Mann-Whitney U test (**c - e**) and two-sided Welch's *t*-test or two-sided Mann-Whitney U test with Bonferroni correction (**f**). Asterisks indicate significant difference, * $p < 0.05$, mean ± s.e.m., circles indicate individual data points. For all, parentheses contain the numbers of animals examined over 2 independent experiments. The *p* values in panel **c – e,** from left to right, are: **c**, *p*=0.02, 0.56, 0.29; **d**, *p*=0.036, 0.66, 0.17; **e**, *p*=0.87, 0.86, 0.88. The Bonferroni corrected *p* value is 1 for every comparison in panel **f**. Source data are provided as a Source Data file.

Supplementary Fig. 13

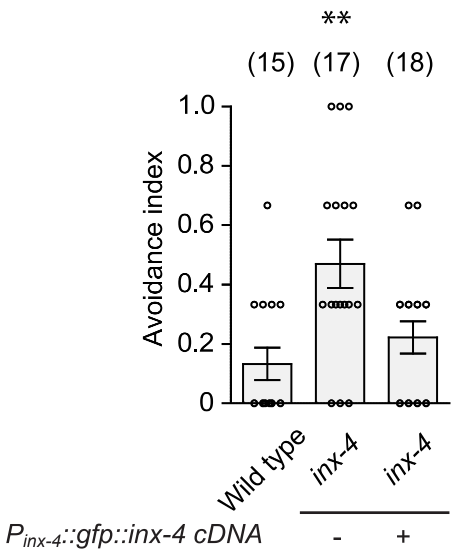

Supplementary Fig. 13. Translational fusion of *gfp::inx-4* rescues hypersensitive response to quinine in the *inx-4* mutants. The avoidance index of 1mM quinine (Methods) is measured in wild type, non-transgenic (-) *inx-4* mutants and transgenic (+) *inx-4* mutants expressing $P_{inx\text{-}4}$::*gfp::inx-4* translational fusion. Animals are compared using Kruskal-Wallis test with Dunn's multiple comparison correction. Asterisks indicate significant difference in comparison with wild type, ** p = 0.0049, mean ± s.e.m.. Parentheses contain the numbers of assays measured over 3 independent experiments and circles indicate individual data points. Source data are provided as a Source Data file.

Supplementary Fig. 14

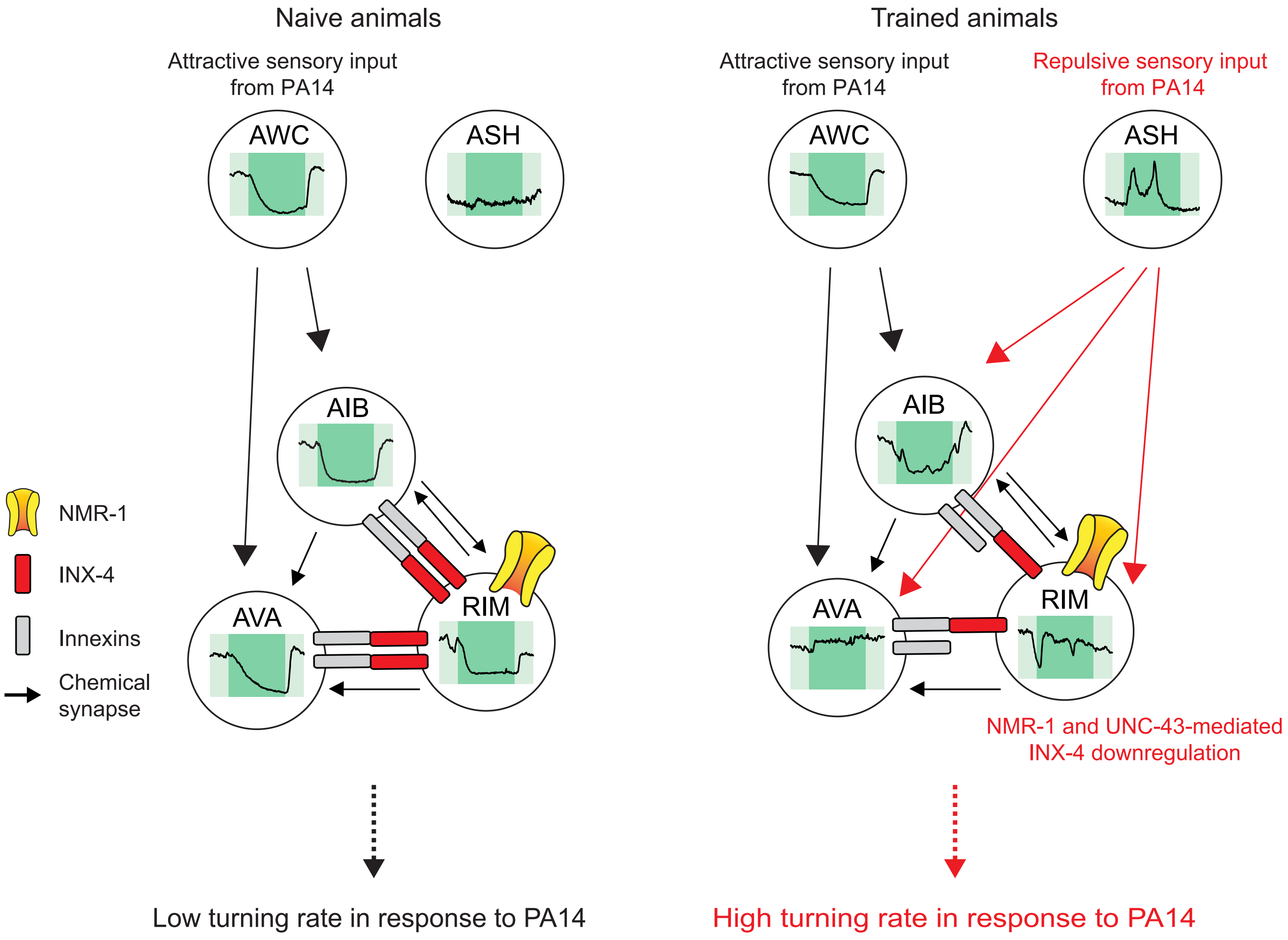

Supplementary Fig. 14. A schematic model showing that training-dependent decoupling of RIM-circuit results from downregulation of gap junction molecule INX-4 in RIM interneurons and training-induced repulsive response to PA14 in ASH sensory neurons, which together reduce the preference for PA14 odorants.

Supplementary Methods

The primers used in this study are:

nmr-1_cDNA-F, 5' TTTTCCTAGGATGTTCCGAATATCAGTTATATTTATTTG 3'
nmr-1_cDNA-R, 5' TTTGGTACCTCACACATAAAATCTAGTTGATCTTG 3'
inx-4_promoter-F, 5' GTAATGTGGGACAAGGTGTACA 3'
inx-4_promoter-R, 5' TCAGAGCAAAGAGCCACTGCT 3'
gcy-13_promoter-F, 5' CTCAAAATCCATTGTGTAAGTTCG 3'
gcy-13_promoter-R, 5' GTCCTGAAAAATTATTGAAAGTTTGTAAT 3'
Cx36_cDNA-F, 5' TTTCGGTACCATGGGAGAGTGGACCATCCT 3'
Cx36_cDNA-R, 5' TTTTGGTACCTTGACGTAGGCGGAGTCGGA 3'
Pnmr-1_loxp-F, 5' CCATGGGTTGTGAAGATGCTT 3'
Pnmr-1_loxp-R, 5' GGATCCTCTAGCGATAACTTC 3'
inx-4_cDNA-F, 5' TTTTGCTAGCATGCCATCTGCTCAACAACACC 3'
inx-4_cDNA-R, 5' TTTTGGTACCCTATACTGCTAAAGGTACATTTTCCATAC 3'
GFP_to_inx-4-F, 5' TTTTGCTAGCATGAGTAAAGGAGAAGAACTTTTC 3'
GFP_to_inx-4-R, 5' TTTTGCTAGCTTTGTATAGTTCATCCATGCCATG 3'
Gibson_backbone_pSM-F, 5' CATGGTATTGATATCTGAGCTCC 3'
Gibson_backbone_pSM-R, 5' GTACCGTCGACGCTAGCC 3'
Gibson_Pgcy-13-F, 5'
GACCCTTGGCTAGCGTCGACGGTACCTCAAAATCCATTGTGTAAGTTC 3'
Gibson_Pgcy-13-R, 5'
TAAACTTGGTGCTTGCGTTCATCATGTCCTGAAAAATTATTGAAAGTTT 3'
Gibson_unc-43_mCherry-F, 5' ATGATGAACGCAAGCACCAA 3'
Gibson_unc-43_mCherry-R, 5'
TGCGGAGCTCAGATATCAATACCATGCTACTTATACAATTCATCCATGCCAC 3'
Q5_unc-43_T286D-F, 5' CAGACAGGATGATGTTGACTGCTTG 3'
Q5_unc-43_T286D-R, 5' TGGATAGCTGATGCAACG 3'
