# Supplementary material for: NMDAR-mediated modulation of gap junction circuit regulates olfactory learning in C. elegans
Source: Nat Commun. 2020 Jul 10;11:3467. doi: 10.1038/s41467-020-17218-0 (PMC7351742; doi:10.1038/s41467-020-17218-0)
Supplement: Supplementary file 3 — Reporting Summary [file 41467_2020_17218_MOESM3_ESM.pdf]

nature research

Corresponding author(s): Yun Zhang

Last updated by author(s): Jun 10, 2020

Reporting Summary

Nature Research wishes to improve the reproducibility of the work that we publish. This form provides structure for consistency and transparency in reporting. For further information on Nature Research policies, see Authors & Referees and the Editorial Policy Checklist .

Statistics

For all statistical analyses, confirm that the following items are present in the figure legend, table legend, main text, or Methods section.

n/a	Confirmed	
☐	☒	The exact sample size (*n*) for each experimental group/condition, given as a discrete number and unit of measurement
☐	☒	A statement on whether measurements were taken from distinct samples or whether the same sample was measured repeatedly
☐	☒	The statistical test(s) used AND whether they are one- or two-sided<br>*Only common tests should be described solely by name; describe more complex techniques in the Methods section.*
☐	☒	A description of all covariates tested
☐	☒	A description of any assumptions or corrections, such as tests of normality and adjustment for multiple comparisons
☐	☒	A full description of the statistical parameters including central tendency (e.g. means) or other basic estimates (e.g. regression coefficient) AND variation (e.g. standard deviation) or associated estimates of uncertainty (e.g. confidence intervals)
☐	☒	For null hypothesis testing, the test statistic (e.g. *F*, *t*, *r*) with confidence intervals, effect sizes, degrees of freedom and *P* value noted<br>*Give P values as exact values whenever suitable.*
☒	☐	For Bayesian analysis, information on the choice of priors and Markov chain Monte Carlo settings
☒	☐	For hierarchical and complex designs, identification of the appropriate level for tests and full reporting of outcomes
☒	☐	Estimates of effect sizes (e.g. Cohen's *d*, Pearson's *r*), indicating how they were calculated

Our web collection on statistics for biologists contains articles on many of the points above.

Software and code

Policy information about availability of computer code

Data collection	NIS-Elements (version AR 4.13.04) imaging software was used to collect data in imaging experiments. The software for behavioral analysis was previously published in Ref 36.
Data analysis	ImageJ v1.50b imaging processing package was used to analyze imaging results. GraphPad Prism v8.4.2 was used for data analysis.

For manuscripts utilizing custom algorithms or software that are central to the research but not yet described in published literature, software must be made available to editors/reviewers. We strongly encourage code deposition in a community repository (e.g. GitHub). See the Nature Research guidelines for submitting code & software for further information.

Data

Policy information about availability of data

All manuscripts must include a data availability statement. This statement should provide the following information, where applicable:

- Accession codes, unique identifiers, or web links for publicly available datasets
- A list of figures that have associated raw data
- A description of any restrictions on data availability

All data generated or analyzed during this study is included in the article and its supplementary information files. The source data underlying Figs 1 -7 and Supplementary Figs 3 -13 are provided as a Source Data file.

Field-specific reporting

Please select the one below that is the best fit for your research. If you are not sure, read the appropriate sections before making your selection.

Life sciences study design

All studies must disclose on these points even when the disclosure is negative.

Sample size	Samples sizes were determined based on previous experiments published in Ref. 23, Ref. 26 and Ref. 43.
Data exclusions	Experiments were not continued if the naive and/or training plates were contaminated with bacteria other than the bacteria designed to use or if the bacterial cultures did not grow normally, because these are critical experimental conditions. This criterion is pre-established. These experiments are not data exclusions. We report this for clarity.
Replication	The numbers of replications are included in all of the figure legends. All attempts at replication were considered successful.
Randomization	Animals were randomly used from the plates where they were grown or trained, and were tested in parallel with appropriate controls.
Blinding	For behavioral experiments, the worms were automatically recorded and analyzed by machine vision softwares. For imaging experiments, the imaging data were recorded, processed and analyzed by commercial softwares. The role of the experimenters is minimal in our experiments.

Reporting for specific materials, systems and methods

We require information from authors about some types of materials, experimental systems and methods used in many studies. Here, indicate whether each material, system or method listed is relevant to your study. If you are not sure if a list item applies to your research, read the appropriate section before selecting a response.

Materials & experimental systems

n/a	Involved in the study	
☒	☐	Antibodies
☒	☐	Eukaryotic cell lines
☒	☐	Palaeontology
☐	☒	Animals and other organisms
☒	☐	Human research participants
☒	☐	Clinical data

Methods

n/a	Involved in the study	
☒	☐	ChIP-seq
☒	☐	Flow cytometry
☒	☐	MRI-based neuroimaging

Animals and other organisms

Policy information about studies involving animals; ARRIVE guidelines recommended for reporting animal research

Laboratory animals	The adult Caenorhabditis elegans hermaphrodites of the following strains were used in this study. N2, CX14996 kyEx4965 [Pinx-1::GCaMP3, Ptdc-1::GCaMP3, Prig-3::GCaMP3, Punc-122::dsRed], RB1834 inx-4(ok2373)V, VM487 nmr-1(ak4)II, ZC2787 nmr-1(ak4)II; kyEx1440[Pgcy-13::nmr-1cDNA, Punc-122::gfp], JN1713 peIs1713[Psra-6::mCasp-1; Punc-122::mCherry], ZC2671 yxEx1371[Podr-1::GCaMP6s, Pstr-2::mCherry, Punc-122::gfp], ZC2905 yxEx1508[Psra-6::GCaMP6s, Punc-122::dsRed], ZC2788 nmr-1(ak4)II; inx-4(ok2373)V, ZC2947 yxEx1531[Pgcy-13::Cx36::mCherry, Pnmr-1s::LoxPSTOPLoxP::Cx36::mCherry, Pflp-18::nCre, Pinx-1::Cx36::mCherry, Punc-122::gfp], ZC2948 yxEx1532[Pgcy-13::Cx36::mCherry, Pinx-1::Cx36::mCherry, Punc-122::gfp], ZC2950 yxEx1534[Pgcy-13::Cx36::mCherry, Pnmr-1s::LoxPSTOPLoxP::Cx36::mCherry, Pflp-18::nCre, Punc-122::gfp], ZC2952 nmr-1(ak4)II; kyEx4965, ZC2953 nmr-1(ak4)II; inx-4(ok2373)V; kyEx4965, ZC2954 yxEx1531; kyEx4965, ZC2955 nmr-1(ak4)II; yxEx1440; kyEx4965, ZC2956 yxEx1536[Pinx-4::gfp::inx-4 cDNA(isoform c), Pgcy-13::mCherry, Punc-122::gfp], ZC2957 nmr-1(ak4) II; yxEx1536, ZC2958 yxEx1537[Pinx-4::gfp, Pgcy-13::mCherry, Punc-122::gfp], ZC2959 nmr-1(ak4)II; yxEx1538[Pgcy-13::unc-43 (T286D)::mCherry, Punc-122::dsRed], ZC2960 yxEx1536; yxEx1538, ZC3326 inx-4(ok2373)V; yxEx1536, ZC3327 nmr-1(ak4)II; inx-4 (ok2373)V; kyEx4965; yxEx1535[Pgcy-13::inx-4cDNA, Punc-122::gfp], ZC3328 peIs1713; yxEx1536. This information is also included in Methods.
Wild animals	The study did not involve wild animals.
Field-collected samples	The study did not involve samples collected from field.
Ethics oversight	The study uses the worm C. elegans, which does not require ethical approval or guidance.

Note that full information on the approval of the study protocol must also be provided in the manuscript.
